# Sporadic Kaposi Sarcoma Following a COVID-19 Vaccine: Mere Coincidence or Something More?

**DOI:** 10.7759/cureus.53925

**Published:** 2024-02-09

**Authors:** Jesús Iván Martínez-Ortega, Arely Gissell Ramirez Cibrian, Elvis Martinez-Jaramillo, Maria del Consuelo García Silva

**Affiliations:** 1 Dermatology, Dermatological Institute of Jalisco, “Dr. José Barba Rubio”, Guadalajara, MEX; 2 Medical Benefits, Mexican Institute of Social Security, Campeche, MEX; 3 Pathology, Faculty of Medicine and Health Sciences, McGill University, Montreal, CAN

**Keywords:** kaposi tumor, sars-cov-2, carcinogen, astrazeneca, vaccine, covid-19, kaposi sarcoma

## Abstract

In this case report, we present a distinctive occurrence of classic Kaposi sarcoma (KS) in an individual of Latin origin, emerging seven days following the administration of the third dose of the ChAdOx1 nCoV-19 (AstraZeneca) vaccine. The progression of KS continued over two months, culminating in the development of a tumor. Given the absence of prior reports on KS development post-COVID-19 vaccination, the primary aim of this report is to explore the potential relationship between the ChAdOx1 nCoV-19 vaccine, reactivation of Kaposi sarcoma-associated herpes virus, and the subsequent onset of KS.

## Introduction

Kaposi sarcoma (KS), in its classic form, was first described by Moritz Kaposi in 1872 and typically presents as sporadic KS, with no history of HIV or men-to-men sexual contact, primarily affecting the skin on the legs. This form is commonly seen in older men of Mediterranean descent, especially Ashkenazi Jews. However, recent attention has brought to light its occurrence in individuals of Latin American origin and is characterized by an indolent, protracted clinical course [1,2]. However, deviating from the typical scenario, our case report brings to attention a unique occurrence, a distinctive case of classic KS in an individual of Latin origin, the progression of KS over two months, which ultimately led to the development of a tumor. With no precedent for KS development post-coronavirus disease 2019 (COVID-19) vaccination, our primary objective is to describe and explore the potential relationship between the ChAdOx1 nCoV-19 vaccine, the reactivation of Kaposi sarcoma-associated herpes virus (KSHV), and the subsequent onset of KS. This unique case challenges the conventional understanding of KS, adding a new dimension to the evolving discourse on vaccine-associated outcomes. This article was previously presented as a poster abstract in 2022 at the Congress of European Academy of Dermatology and Venereology.

## Case presentation

**Figure 1:**
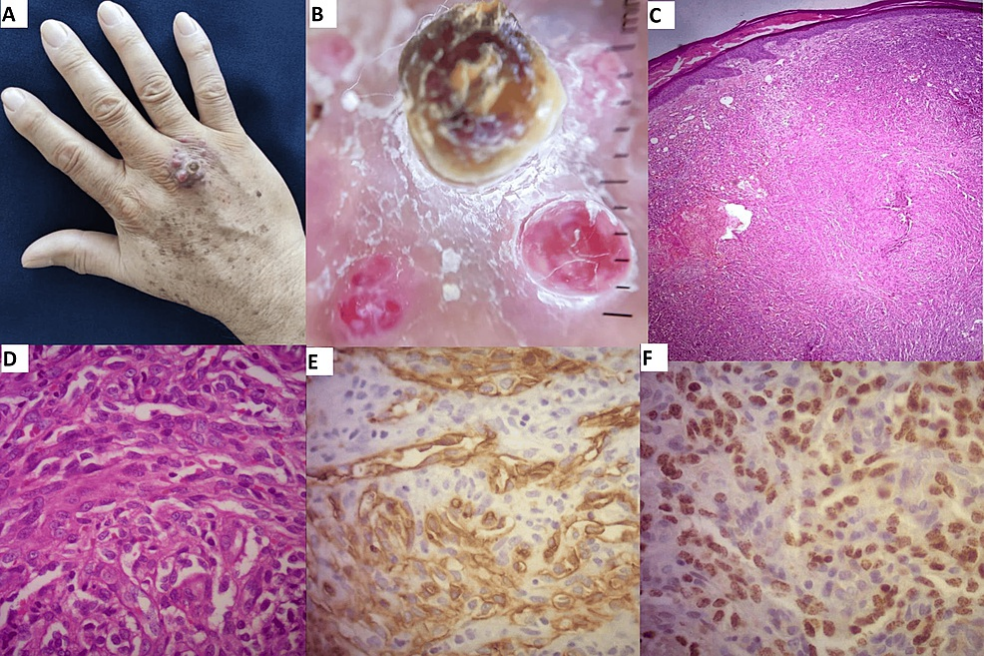
Panel (A) clinical image of the lesion, (B) dermoscopy image of the lesion, (C) hematoxylin and eosin stain showing a vascular tumor invading the dermis composed of a proliferation of spindle-shaped cells arranged in irregular and slit-like vascular spaces. Extravasated red blood cells can be observed within the vascular spaces, at 10x and (D) 40x magnification. (E and F) Immunohistochemistry staining for CD34 and HHV8 LANA (latency-associated nuclear antigen) respectively.

We present the case of a 73-year-old man who visited the dermatology clinic with a 2×3x1 cm infiltrated plaque and multiple violaceous and red nodules on the dorsal aspect of his right hand (Figure 1).

The lesions had been noticed for two months and were asymptomatic. The patient had a medical history of dyslipidemia and arterial hypertension. He reported that the lesion appeared one week after receiving the third dose of the COVID-19 vaccine (ChAdOx1 nCoV-19), gradually growing over the course of two months until reaching its current appearance. The patient lacks Mediterranean ancestry, did not use any immune-modulating agents, denied any prior COVID-19 infection, and reported no engagement in sexual activity with men. Regarding his general health, both subjectively and objectively, there were no reported compromises or significant effects. HIV testing yielded negative results and histopathological examination confirmed the diagnosis of classic KS through positive immunohistochemistry analysis for CD34 and HHV-8. Cryotherapy was selected as the treatment approach, achieving full clearance after three sessions, spaced three weeks apart for each session.

## Discussion

We conducted a thorough search on PubMed and Google Scholar using the keywords 'Kaposi sarcoma' or 'Kaposi tumor' in combination with 'vaccine' and 'COVID-19.' Despite our efforts, we found no articles linking KS to any COVID-19 vaccination. While the strong epidemiological correlation makes it less likely for other factors to be the cause, the absence of a background compelled us to speculate on potential mechanistic theories. KSHV is an absolute requirement of oncogenesis and is a direct carcinogen, so when we are looking into the relationship between the AstraZeneca vaccine and Kaposi sarcoma, we may focus on the reactivation of KSHV [1].

Studies have shown that spike proteins of SARS-CoV-2 can reactivate the lytic phase of KSHV. The ChAdOx1 nCoV-19 vaccine contains DNA eDNA-encoding proteins. If these spike proteins encounter HHV8-infected cells, it could potentially trigger the reactivation of the virus, leading to the lytic phase [3].

**Figure 2:**
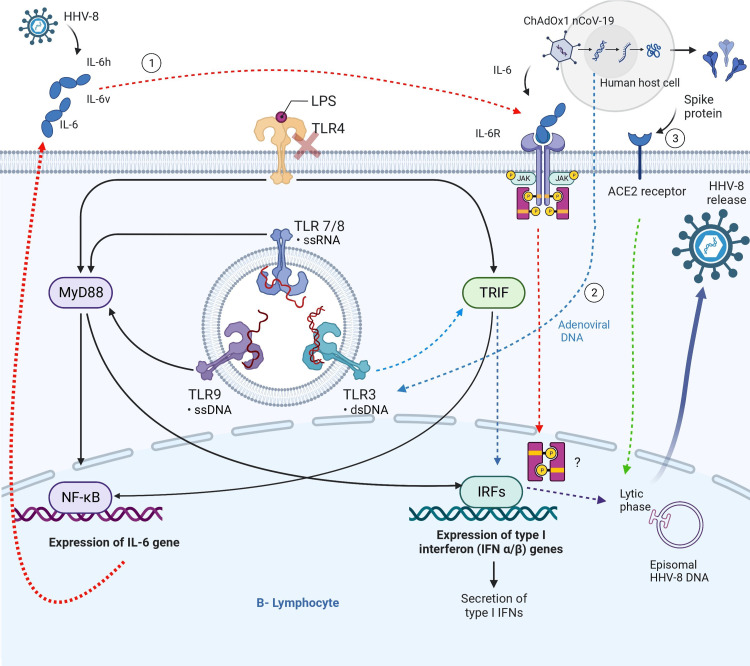
Hypothesized mechanisms underlying reactivation of HHV-8 by the ChAdOx1 nCoV-19 vaccine. (1) Human herpes virus 8 (HHV-8) engages the immune system, leading to the synthesis of human interleukin 6 (IL-6h) by the host and viral interleukin 6 (IL-6v) produced from viral DNA. The ChAdOx1 nCoV-19 vaccine stimulates the production of interleukin-6 (IL-6). The Toll-like receptor (TLR) pathway facilitates the production of interleukin-6 (IL-6) by activating nuclear factor-kappa B (NF-kB) transcription factors through the involvement of MyD88 and TRIF. Interleukin-6 (IL-6) forms a complex with its specific receptor, initiating the activation of the lytic phase of Human Herpes virus 8 (HHV-8). Nevertheless, the mechanism of this activation via the JAK/STAT3 pathway remains speculative and lacks conclusive evidence. Adenoviral DNA or RNA transcripts have the potential to stimulate intracellular Toll-like receptors (TLRs) 3, 7, 8, and 9. This activation, facilitated by TRIF and IRFs, leads to the initiation of the HHV-8 lytic phase. The binding of spike proteins to the ACE receptor results in the reactivation of the lytic phase of HHV-8. ACE receptor (angiotensin-converting enzyme), HHV-8 (herpes virus 8), interferon-regulatory factor, IL-6h (human Interleukin 6) IL-6v (viral IL-6), LPS (Lipopolysaccharides), MyD88 (Myeloid differentiation primary response 88), TLR (toll-like receptor), TRIF (TIR-domain-containing adapter-inducing interferon-β). Created with Biorender.

The reactivation of KSHV may also be influenced more by the immune inflammation stimulated by the adenovirus vector rather than the spike proteins themselves (Figure 2).

There are two main pathways through which this reactivation may occur. Firstly, both human IL-6 and the KSHV-encoded homolog vIL-6 have been shown to reactivate KSHV [4]. Studies have demonstrated that adenovirus vector infection of human epithelial cells induces the expression of pro-inflammatory cytokines, particularly IL-6. This activation is αvβ3 integrin-dependent and NF-κB mediated. Additionally, αvβ3 integrin has been found to be involved in the angiogenic pathogenesis of Kaposi sarcoma, induced by vIL-6 [5,6]. Therefore, we believed that the IL-6 (and vIL-6 after reactivation of KSHV) induced by adenovirus vector vaccination may reactivate KSHV and potentially contribute to the angiogenesis and oncogenesis processes through αvβ3 integrin expression.

Secondly, since the adenovirus vector contains DNA strands, it could stimulate the reactivation of KSHV through the Toll-like receptor (TLR) 7/8 intracellular receptors in various cells. Although one might expect adenoviral vector infection to activate TLR 4, which is an upstream pathway for IL-6, studies have shown that TLR 4 activation does not reactivate KSHV [7].

Regarding the mechanism of vaccine distribution to distant tissues, while spike proteins and mRNA templates can spread systemically after intramuscular administration, cytokines typically exert more localized and paracrine effects [8]. However, studies have observed an increase in plasma IL-6 levels following COVID-19 vaccination [9]. Therefore, all three possibilities, spike protein-mediated reactivation, adenovirus-induced cytokine IL6, and TLR7/8 activation by DNA, remain plausible hypotheses to explain the development of KS on the dorsum of the hand in this case. In addition, the formation of immune cell syncytia induced by the SARS-CoV-2 spike protein has been associated with oncogenesis. Our theory posits that the increased fusion of nuclei from different lymphocytes (CD45) raises the probability of KSHV DNA interaction, especially if any of the cells were already infected [10]. Therefore, we propose that syncytia formation triggered by the spike protein might function as a mechanism for reactivating and propagating KSHV. To the best of our knowledge, this is the first reported case of KS associated with the SARS-CoV-2 vaccine.

## Conclusions

This case highlights a distinctive occurrence of classic KS in an individual of Latin descent, emerging shortly after the administration of the ChAdOx1 nCoV-19 vaccine. While more research is needed to establish a definitive connection, the evidence discussed in this report points to potential mechanisms involving KSHV reactivation, the influence of adenovirus-induced inflammation, and spike protein-related effects. Emphasizing the need for ongoing investigation, this case underscores the importance of understanding the interplay between COVID-19 vaccines, KSHV, and the development of KS. Continued research and vigilance are crucial for advancing our knowledge of KSHV oncogenesis and its potential relationship with vaccinations.
